# Sensor Fusion Pipeline for Gait Analysis Using Insole-Embedded Inertial Measurement Units (IMUs)

**DOI:** 10.3390/s26217029

**Published:** 2026-11-06

**Authors:** Juliette Ngan, Yuanwei Liu, Ali Asgar Saleem Bhagat

**Affiliations:** 1 Institute for Health Innovation & Technology (iHealthtech), National University of Singapore (NUS), MD6, 14 Medical Drive, #14-01, Singapore 117599, Singapore; 2 Department of Biomedical Engineering, National University of Singapore (NUS), 4 Engineering Drive 3, Singapore 117583, Singapore

**Keywords:** gait analysis, wearable sensors, inertial measurement unit (IMU), smart insole, spatiotemporal gait parameters, sensor fusion

## Abstract

Wearable gait assessment using foot-mounted inertial measurement units (IMUs) can extend mobility evaluation beyond the laboratory, but performance is limited by integration drift, magnetic disturbances, and gait event detection sensitivity during outdoor overground walking. This study presents an insole-embedded nine-axis IMU pipeline combining orientation estimation using the Versatile Quaternion Filter with zero-velocity update-aided error-state Kalman filtering and Rauch–Tung–Striebel smoothing. Twenty-one healthy adults performed slow, normal, and fast 100 m straight path walks and normal-speed 437.7 m full-lap walks on an outdoor track. Seventy-five retained trials were compared with the Movella MVN Awinda reference using task-specific absolute-agreement intraclass correlation coefficients (ICCs) and mixed-effects Bland–Altman analysis accounting for repeated observations within participants. Agreement was excellent for stride length and stride time (ICCs: 0.928–0.961 and 0.989–0.997, respectively), good to excellent for gait velocity (0.894–0.957), moderate to excellent for stance time (0.679–0.942), and moderate to good for swing time (0.544–0.790). Mean biases were +0.036 m/s for gait velocity and +1.54 cm for stride length; temporal biases ranged from −0.005 to +0.005 s. Walking distance mean absolute percentage errors ranged from 0.55% to 1.20%. These findings support the feasibility of insole-based spatiotemporal gait assessment during predefined outdoor walking in healthy adults. Further validation against independent reference systems and in clinical populations and more complex environments is needed.

## 1. Introduction

Gait is widely recognized as an important indicator of health and an essential component of mobility assessment [1,2,3]. It can be used to monitor disease progression [1,4,5], aid diagnosis [5,6], and guide rehabilitation [1,4,6]. It also has applications in monitoring exertional heat stroke [7], fatigue [7,8], and workplace safety and performance [9].

Spatiotemporal gait parameters, such as gait velocity, stride length, stride time, stance time, and swing time, are used in quantitative gait evaluation. They capture characteristic changes in walking function associated with neurological and musculoskeletal conditions, including reduced stride length in Parkinson’s disease [10], step length reductions in progressive multiple sclerosis [11], and stance–swing timing asymmetries in post-stroke gait [12]. Stride-to-stride variability in timing and length has additionally been linked to fall risk in older adults [13,14]. Among these parameters, gait velocity is often regarded as a functional “vital sign” [15]: it is associated with adverse outcomes and survival in older adults [3,16] and may carry prognostic value in contexts such as post-stroke recovery [17] and fracture healing [18].

Quantitative gait assessment typically relies on laboratory-based motion capture systems, which provide high-fidelity 3D kinematics and remain a common reference for validation studies [4,19,20,21]. Despite their high accuracy, these systems are expensive and typically capture short supervised walking bouts, which may not reflect daily mobility in natural environments [2,22]. This gap has accelerated interest in wearable gait assessment.

In particular, inertial measurement unit (IMU)-based systems, enabled by low-cost sensors, have gained popularity. These sensors can be deployed beyond the lab to support repeated assessments and even continuous monitoring in home, community, and workplace settings. IMUs commonly comprise a three-axis accelerometer (specific force) and a three-axis gyroscope (angular velocity). They may additionally incorporate a three-axis magnetometer (local magnetic field vector components), resulting in a nine-axis configuration. The magnetometer provides a reference to the Earth’s magnetic field, enabling the estimation and correction of heading. In principle, simply integrating these signals yields orientations, velocities, and displacements, from which spatiotemporal gait parameters can be derived. However, in practice, sensor bias, noise, magnetic interference [23,24], and the sensor position [25,26] introduce errors that accumulate over time, particularly in the absence of an absolute position reference (e.g., GPS), eventually rendering the trajectory estimate unreliable [27].

Various approaches have been proposed to estimate gait parameters while avoiding or mitigating these limitations. The gait cycle is segmented by detecting gait events, primarily initial contact and toe-off, whose timing directly yields temporal parameters such as stride and swing time. Detection methods range from rule-based techniques that use characteristic signal features such as peaks and zero-crossings to model-based approaches that impose biomechanical constraints on the gait cycle and to machine learning methods [28]. The estimation of spatial parameters is commonly achieved through the direct integration of inertial signals, with gait-specific constraints applied to limit the accumulated drift [29,30]. Alternative strategies include biomechanical models, notably the inverted pendulum model applied to trunk-mounted sensors [30,31], and data-driven machine learning approaches that learn mappings from sensor signals to gait parameters using reference measurements [32,33]. Biomechanical models are sensitive to assumptions about gait mechanics, while machine learning methods require representative training data and may not generalize across populations or conditions.

In this work, we adopt the integration-based strategy, combining rule-based gait event detection with drift-corrected trajectory reconstruction to estimate spatiotemporal gait parameters. The integration-based approach introduces two technical requirements: accurate orientation estimation, needed to resolve sensor-frame accelerations into a global reference frame, and effective drift mitigation during the double integration of the resulting gravity-compensated acceleration. For orientation, complementary filters such as the Mahony and Madgwick filters are widely used due to their simplicity and low computational cost [34,35]. However, these filters rely on the accelerometer as a gravity reference for inclination correction, and their heading estimates are vulnerable to local magnetic field disturbances. We therefore implement the Versatile Quaternion-Based Filter (VQF), which is more robust to both effects [36]. For drift mitigation, we apply a zero-velocity update (ZUPT)-aided error-state Kalman filter, which uses stance-phase zero-velocity constraints to limit position errors [29,37]. Residual discontinuities at ZUPT correction boundaries are then reduced by Rauch–Tung–Striebel (RTS) smoothing [38].

We propose a comprehensive pipeline for estimating spatiotemporal gait parameters from a pair of insole-embedded nine-axis IMU sensors. The pipeline is implemented within the Gaitmap ecosystem, an open-source suite for foot-mounted IMU-based gait analysis [39]. Our contributions are threefold. First, we introduce a coupled orientation and trajectory estimation framework that combines the Versatile Quaternion-Based Filter [36] for orientation estimation with a ZUPT-aided error-state Kalman filter (ESKF) and RTS smoothing for trajectory reconstruction [38,39,40]. Second, we provide experimental validation for outdoor overground walking conditions across multiple speeds and distances—conditions that impose greater demands on drift mitigation than the short indoor walkways typical of most validation studies. Third, we benchmark the proposed pipeline against the Movella MVN Awinda reference system, quantifying measurement agreement for key spatiotemporal gait parameters.

## 2. Methodology

### 2.1. Participants

For our experiment, a total of 21 participants (9 female, 12 male), aged between 21 and 43 years, were recruited. Participants were excluded if they had physical impairments or injuries that could affect gait or sensor use or chronic medical conditions that could limit their physical fitness. All procedures were explained, and written informed consent was obtained before participation. Demographics were recorded as follows (mean ± standard deviation (SD)): age 27.6 ± 5.8 years; height (with shoes) 172.0 ± 7.4 cm; shoe length 28.9 ± 1.7 cm. All participants wore their own trainers with laces. The study was approved by the Institutional Review Board of the National University of Singapore (NUS-IRB-2024-690).

### 2.2. Experimental Set-Up

#### 2.2.1. IMU Insoles

**Figure 1:**
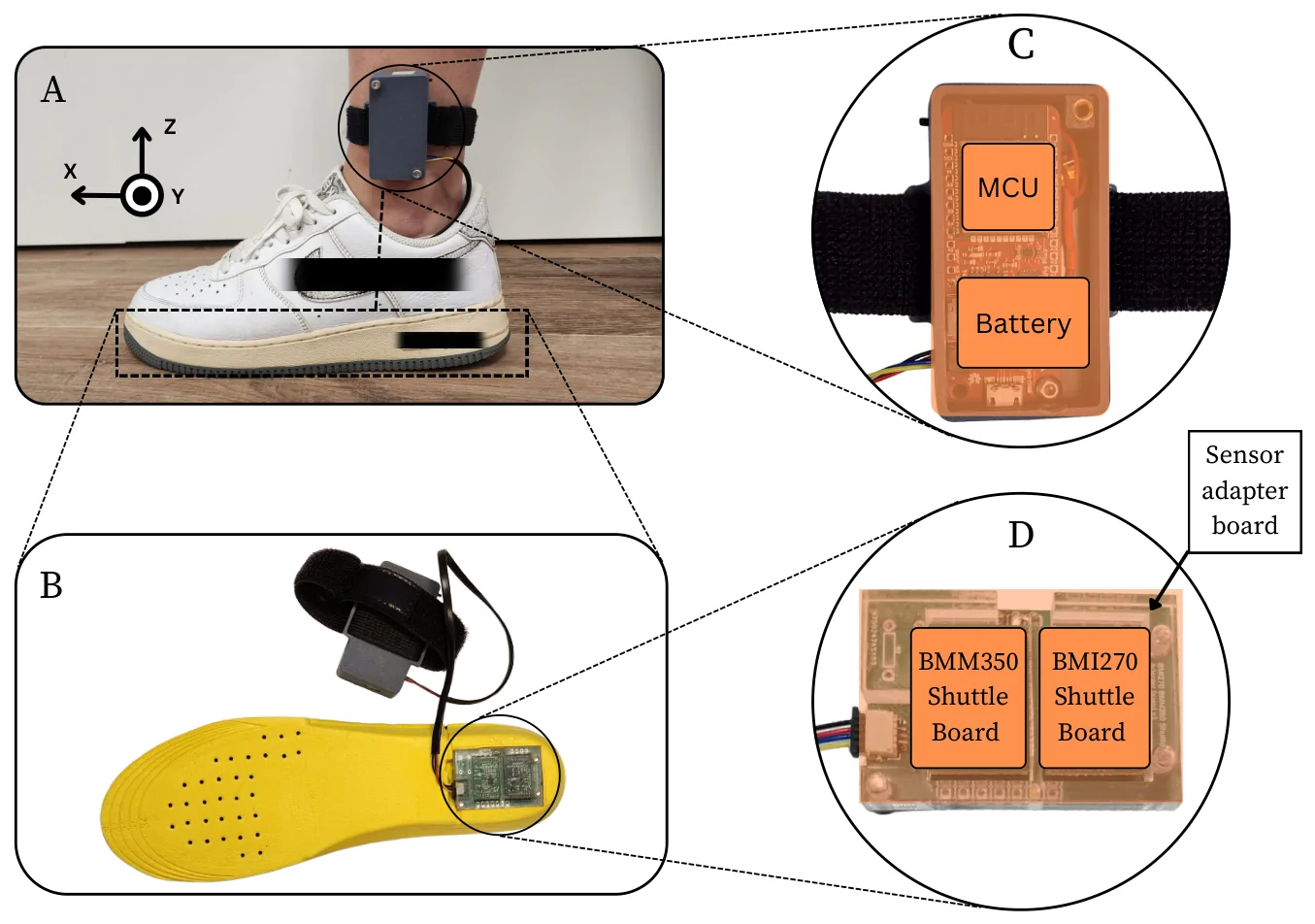
IMU insole hardware. (**A**) Left foot configuration showing the insole cable connected to the ankle-mounted unit. (**B**) Modified insole with an internal cavity housing the embedded IMU sensor module, connected to the ankle unit. (**C**) Ankle unit (ESP32 and battery) with a strap for securing to the ankle; this unit performs data acquisition and wireless transmission. (**D**) IMU sensor module comprising a BMI270 shuttle board (gyroscope and accelerometer) and a BMM350 shuttle board (magnetometer) mounted on a sensor adapter board.

Custom insoles were created by modifying high-density foam insoles to include a cavity that housed a removable sensor module, positioned approximately 1 cm from the back edge of the insole (Figure 1B). Three base insole sizes were used and trimmed to each participant’s shoe length to provide an appropriate fit within their footwear. The module contained a BMI270 accelerometer/gyroscope and a BMM350 magnetometer (Bosch Sensortec, Reutlingen, Germany) (Figure 1D). The sensor module was connected by a cable to an ankle-mounted control unit (Figure 1B) that housed an ESP-32 Thing Plus micro-controller (SparkFun Electronics, Niwot, CO, USA), powered by a 3.7 V, 600 mAh lithium-ion battery (Figure 1C).

Sensor data were acquired at 100 Hz, with an accelerometer range of ±8 g, a gyroscope range of ±2000 °/s, and a magnetometer range of ±2000 µT. All data were transmitted via Bluetooth and logged on a laptop for offline processing.

#### 2.2.2. MVN Awinda Reference System

**Figure 2:**
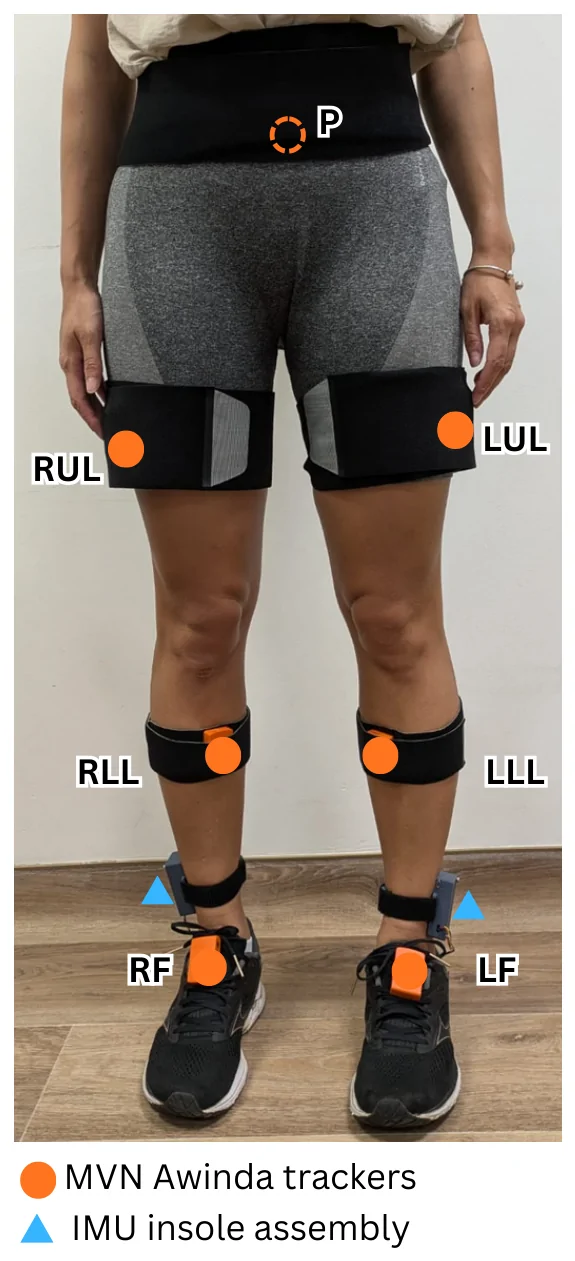
Full experimental setup on a participant. Seven MVN Awinda inertial trackers (orange) were placed on the lower limbs and trunk, together with two custom IMU insole assemblies (blue; left and right), to record lower body and foot motion simultaneously. MVN Awinda tracker locations are labeled as P (pelvis), RUL/LUL (right/left upper leg), RLL/LLL (right/left lower leg), and RF/LF (right/left foot). The pelvis tracker is positioned posteriorly and is indicated by a dashed circle, as it is not visible in the frontal view.

The MVN Awinda IMU motion capture system (Movella Inc., Henderson, NV, USA) was used as the reference measurement system. Although optical motion capture is widely regarded as the laboratory gold standard, it is impractical for outdoor, real-world validation. Therefore, MVN Awinda was selected to match the intended deployment environment of our pipeline. Following MVN Awinda’s lower-limb motion capture configuration, participants wore seven MTw Awinda motion tracker units (Figure 2). Movella’s cloud-based MVN Gait Report software was used to derive spatiotemporal gait parameters. The validity of gait parameters derived by the MVN Gait Report has previously been evaluated against a synchronized optical motion capture reference system [41]. For each recording, parameters are reported separately for each foot and summarized as the mean ± SD.

### 2.3. Experimental Protocol

**Figure 3:**
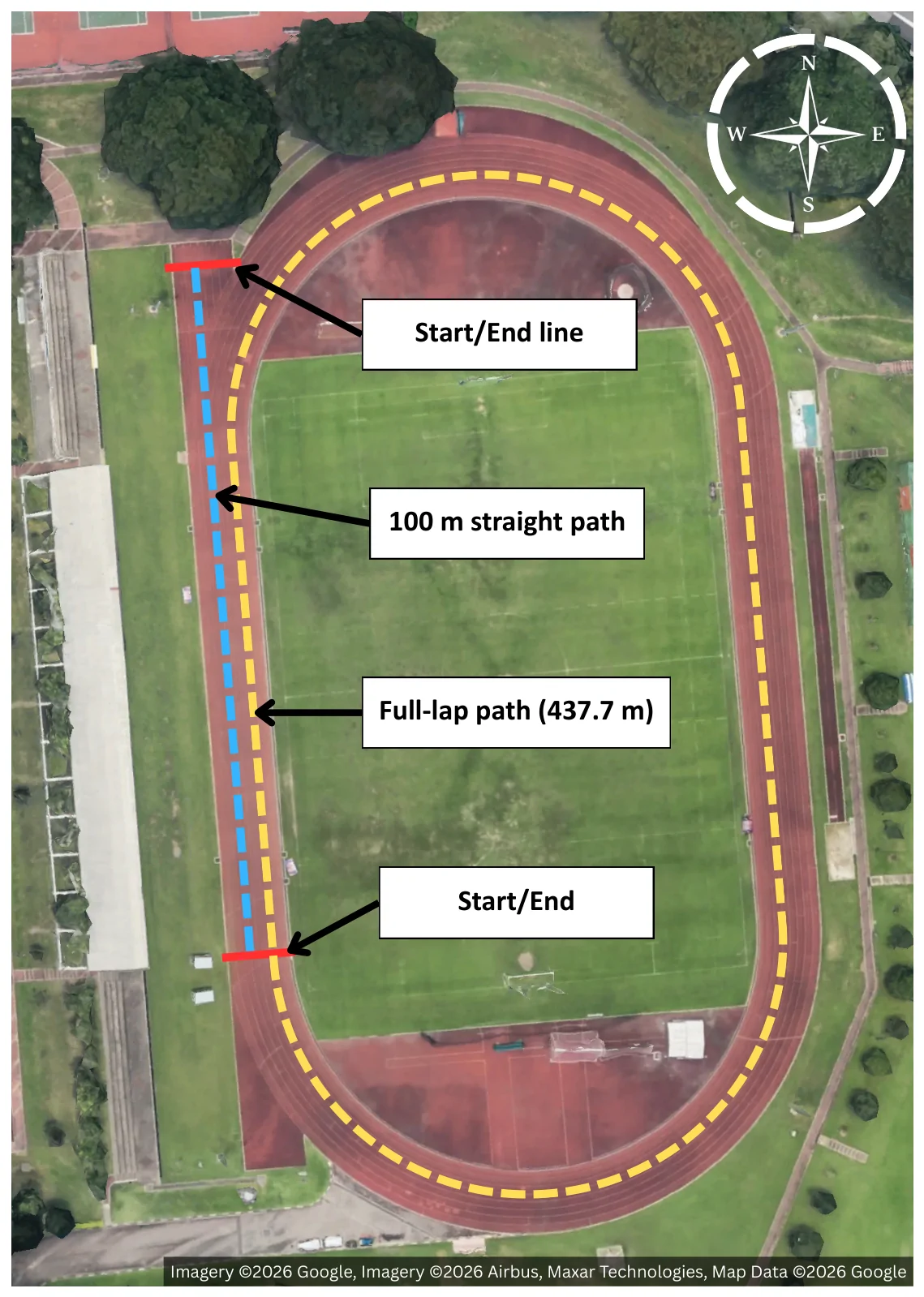
Walking paths used in the experimental protocol. Aerial view from Google Maps LLC [43] (accessed on 22 January 2026) of the 400 m running track at the National University of Singapore, showing the 100 m straight walking paths on the track lane (blue) and the 437.7 m full-lap walking path around the track (yellow). Red lines indicate the common start/end line for all straight-path trials. These paths correspond to tasks T1–T4 in Table 1.

The trials were carried out on the 400 m running track at the National University of Singapore, with all trials conducted under dry ground conditions. Prior to data collection, a short calibration procedure was performed for the MVN Awinda system according to the software’s guided steps [42]. As described in Table 1 and illustrated in Figure 3, each participant completed one trial of each of four walking tasks in sequence: normal-speed 100 m straight walking (T1), slow 100 m straight walking (T2), fast 100 m straight walking (T3), and normal-speed full-lap walking (T4). The full-lap route was measured with a measuring wheel at 437.7 m along the outer line of lane 6 and is referred to as “Normal (full lap)” throughout this paper.

### 2.4. Analysis Pipeline

**Figure 4:**
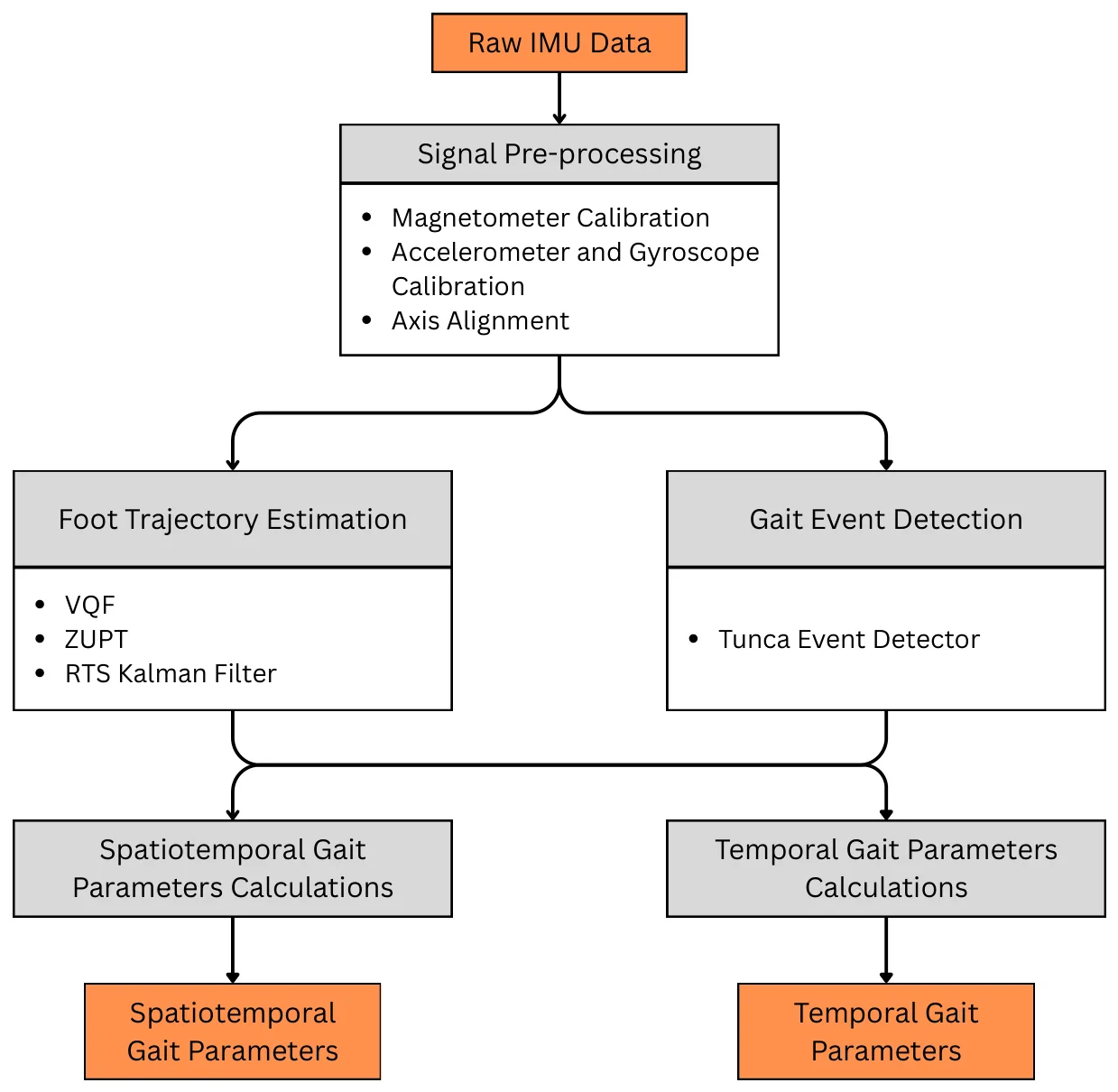
Gait analysis pipeline overview. Raw 9-axis IMU signals from insole-embedded sensors are first pre-processed (accelerometer/gyroscope calibration, magnetometer hard/soft-iron compensation, axis alignment). Two parallel branches follow: (i) orientation estimation and ZUPT-aided trajectory estimation (VQF for attitude coupled with an ESKF for position/velocity; initialized with the initial foot positions) and (ii) gait event detection (heel strike and toe-off). The trajectories and detected events are then used to compute temporal and spatiotemporal gait parameters (e.g., stride length, stance/swing times, and speed).

The gait analysis pipeline was developed to estimate spatiotemporal gait parameters using data from 9-axis IMUs embedded within insoles. Conceptually, the pipeline consists of four main stages: (1) signal pre-processing, (2) gait event detection, (3) foot trajectory estimation, and (4) gait parameter computation (Figure 4). Each component of the pipeline is described in the following subsections.

#### 2.4.1. Signal Pre-Processing

Raw IMU data are first calibrated to correct for sensor bias and scale errors. The calibration coefficients for the gyroscope and accelerometer are determined using the method developed in Küderle et al. [44] and Ferraris et al. [45].

Magnetometer measurements are compensated for hard-iron offsets and soft-iron distortions, as well as sensor scale and cross-axis errors. The correction parameters are estimated from a calibration dataset collected by manually rotating the sensor at the data collection site to cover a broad range of orientations and are obtained via least squares ellipsoid fitting [46,47].

#### 2.4.2. Gait Event Detection

Gait event detection is essential for determining gait parameters. The event detection algorithm used in this work is based on the gyroscope-only approach proposed by Tunca et al. [48] and adopted by Trautmann et al. [49]. In brief, stance phases were identified from periods with a low angular rate, and heel strike (HS)/toe-off (TO) were subsequently located as prominent peaks in the angular velocity within each stride segment.

#### 2.4.3. Foot Trajectory Estimation

Orientation estimation was performed to transform sensor data from the foot’s local reference frame to the North–West–Up global frame (x: North, y: West, z: Up). We use the VQF [36], which estimates the inclination from accelerometer data that are low-pass-filtered in an almost inertial frame to better reject transient non-gravitational accelerations and decouples magnetometer-based heading correction so that magnetic disturbances do not corrupt the tilt estimate. The time constants were set to 1.0 for magnetometer-based heading correction and 5.0 for accelerometer low-pass filtering. In addition, the VQF is computationally efficient and, as a quaternion-based orientation estimation algorithm, avoids the gimbal lock associated with Euler angle representations. After this, the foot velocity and displacement were estimated.

To mitigate sensor drift, a ZUPT scheme with an ESKF incorporating RTS smoothing [38,39,40] was implemented. Stationary periods, treated as zero-velocity pseudo-measurements, are identified when the mean gyroscope magnitude falls below a predefined threshold (34 degrees/s) and are passed to the ESKF to constrain drift. The subsequent RTS smoothing refines the error-state estimates over the entire trial, reducing ZUPT-related discontinuities [38]. In a final correction step, these smoothed errors are applied to correct the position, velocity, and orientation trajectories.

Walking distance was estimated independently for each foot by summing the Euclidean displacement between consecutive horizontal (*x*–*y*) position samples over the trial. The left- and right-foot estimates were averaged to obtain a single distance estimate for each trial.

#### 2.4.4. Spatiotemporal Gait Parameters

**Figure 5:**
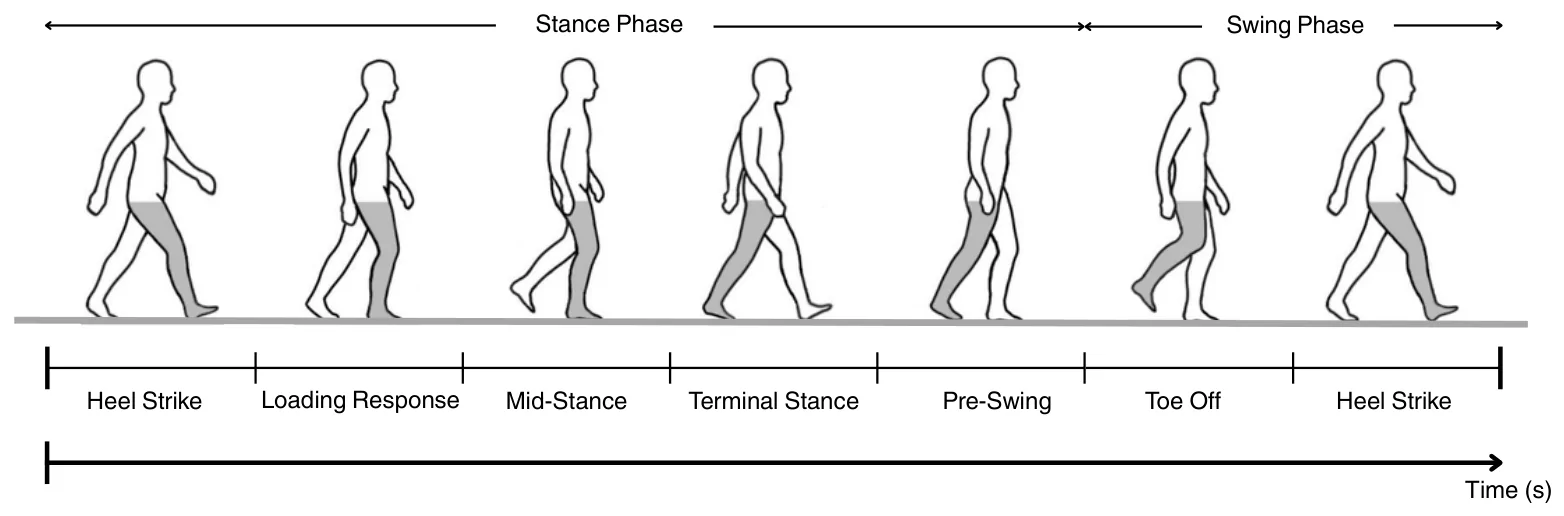
Phases of a normal gait. Illustration of the gait cycle for the leg shown in grey, showing stance and swing phases with key events (heel strike and toe-off) and the corresponding definitions of stance time and swing time.

Once individual strides have been segmented and foot trajectories estimated, spatiotemporal gait parameters are computed. A stride is defined as the gait cycle between two consecutive HS of the same foot (Figure 5). The first and last strides of each gait bout are excluded from analysis due to inconsistent foot placement at gait initiation and termination. The following parameters are computed for each stride:

- Stride time—time between consecutive HS of the same foot;
- Stance time—duration of foot contact with the ground;
- Swing time—time for which the foot is in the air;
- Stride length—total distance covered in one stride;
- Gait velocity—average walking speed, computed per stride as the stride length divided by the stride duration.

Within each trial, we summarized the stride-by-stride parameters for each foot by averaging across all remaining strides. For each parameter, the per-foot mean and SD (across strides) are kept as inputs for subsequent statistical analyses.

### 2.5. Statistical Analysis

The validation of the spatiotemporal gait parameters was performed by comparing the results from tasks T1–T4 derived by our algorithm with the corresponding values reported by the MVN Gait Report. For our pipeline, stride-level values were averaged within each foot, and the two foot-specific means were then averaged equally to obtain one bilateral trial-average observation. For the MVN Gait Report, we used the reported bilateral gait velocity and averaged the left- and right-foot trial means for the stride length, stride time, stance time, and swing time, which were provided from the software. Measurements from both systems were then paired by participant and trial. The analysis included these paired trial-average observations per parameter from all 21 participants.

Using these values, we generated Bland–Altman plots [50] to analyze the agreement between the two methods for the gait velocity, stance time, stride length, stride time, and swing time. For each parameter, the difference between methods (error = our algorithm − MVN Gait Report) was plotted against the mean of the two measurements.

To account for the repeated observations within participants, differences between our algorithm and the MVN Gait Report were modeled as

$$d_{ij}=\mu +u_{i}+\epsilon_{ij},$$

where μ is the pooled mean between-system bias across the four walking tasks, $u_{i}$ is a participant-specific random intercept, and $\epsilon_{ij}$ is the residual discrepancy. The linear mixed-effects model was fitted by restricted maximum likelihood (REML). The 95% limits of agreement (LOAs) were then calculated as

$$\hat{\mu }\pm 1.96\mathrm{SD},$$

where $\hat{\mu }$ is the estimated mean bias and $\mathrm{SD}=\sqrt{{\hat{\tau }}^{2}+{\hat{\sigma }}^{2}}$ is the estimated standard deviation of the between-system differences, incorporating both participant-level random-intercept variance (${\hat{\tau }}^{2}$) and residual variance (${\hat{\sigma }}^{2}$). Wald-type 95% confidence intervals were calculated for the mean bias. The normality of the random intercepts and conditional residuals was assessed using Q–Q plots. The coefficient of variation (CV) was also reported as CV=SD/mean×100%.

Agreement between methods was further quantified using the task-level intraclass correlation coefficient (ICC). For each parameter, we computed a two-way mixed-effects, absolute-agreement, single-measure ICC (ICC(A,1)) [51] between our pipeline and the MVN Gait Report for each of the four walking tasks. Participants were treated as random effects and the two measurement systems as fixed effects. Each analysis included one paired trial-average observation per participant. ICC values were interpreted according to Koo and Li [52] as follows: excellent (>0.90), good (0.75–0.90), moderate (0.50–0.75), and poor (<0.50).

Furthermore, we performed simple ordinary least squares (OLS) regression for each parameter, with the MVN Gait Report value as the independent variable and our algorithm as the dependent variable. We report the regression equation, Pearson correlation coefficient (*r*), and coefficient of determination ($R^{2}$) to describe the pooled linear association. The RMSE was calculated directly from the paired between-system differences. All statistical analyses were performed in Python (version 3.11.5) using NumPy (version 1.24.3) [53], SciPy (version 1.11.1) [54], pandas (version 2.0.3), and statsmodels (version 0.14.0). Plots were generated using Matplotlib (version 3.11.2) [55].

## 3. Results

### 3.1. Data Overview

A total of 84 trials (four per participant) were completed by the 21 participants between December 2024 and July 2025. Five trials were excluded because of incomplete data, and one was excluded because poor Awinda calibration prevented the processing of the MVN Gait Report. Post hoc quality control review identified three additional trials with markedly shortened MVN walking windows: one 100 m fast walking trial covered 48.32 m, while two 437.7 m full-lap trials covered 181.51 m and 47.88 m. These trials were excluded because the MVN Gait Report analyzed much shorter walking segments, raising concerns that the two systems’ trial averages were not directly comparable. The remaining 75 trials from all 21 participants were retained for the primary analyses.

### 3.2. Gait Detection and Distance Estimation

Table 2 summarizes the number of strides detected and the walking distances estimated by the proposed pipeline across the four walking tasks.

### 3.3. Spatiotemporal Gait Parameters: Association and Agreement with MVN Awinda

**Figure 6:**
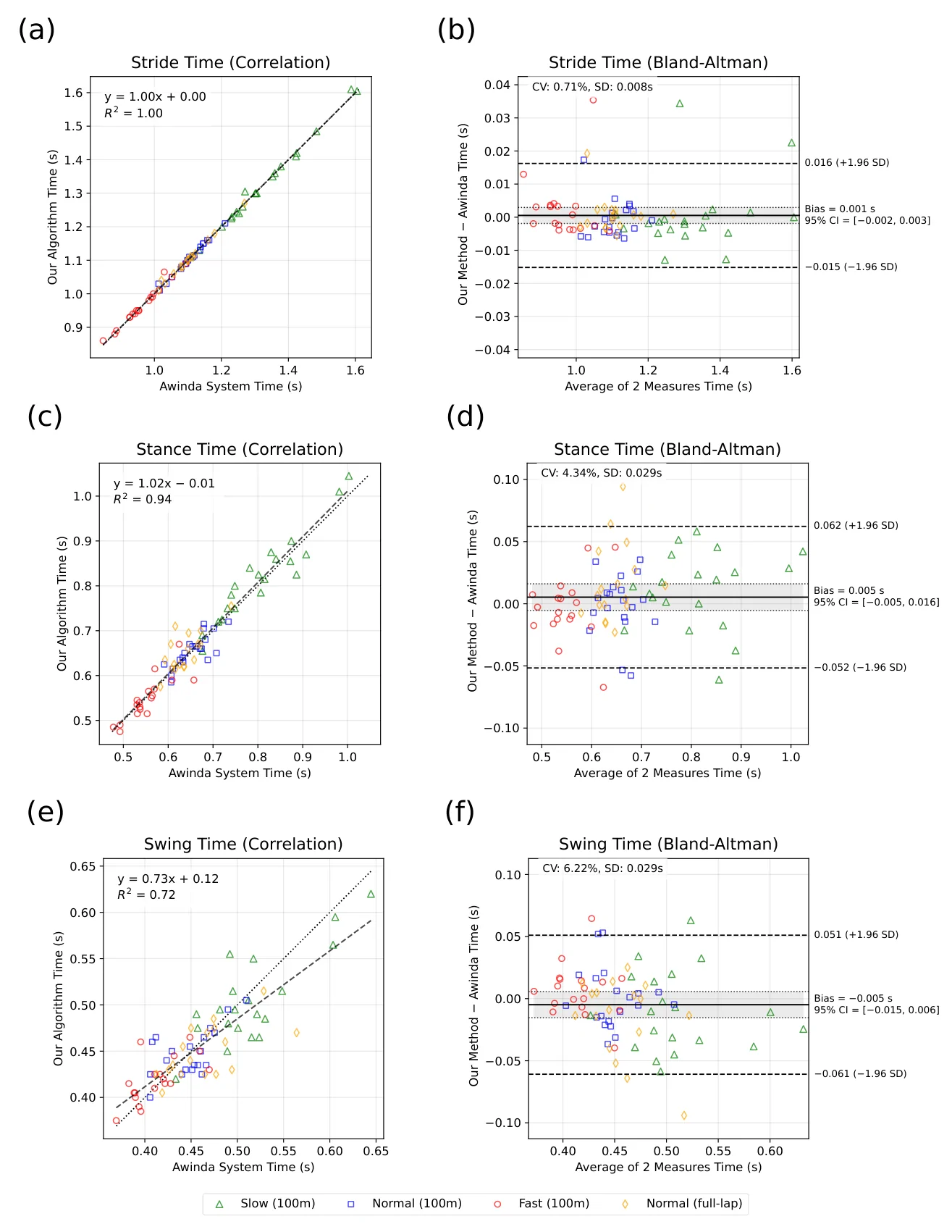
Agreement between our pipeline and the MVN Awinda reference for temporal gait parameters across all trials. Correlation plots are shown in the left column and Bland–Altman plots in the right column. In the correlation plots, the dotted line represents the identity line, and the dashed line represents the fitted regression line. Stride time is shown in (**a**,**b**), stance time in (**c**,**d**), and swing time in (**e**,**f**).

**Figure 7:**
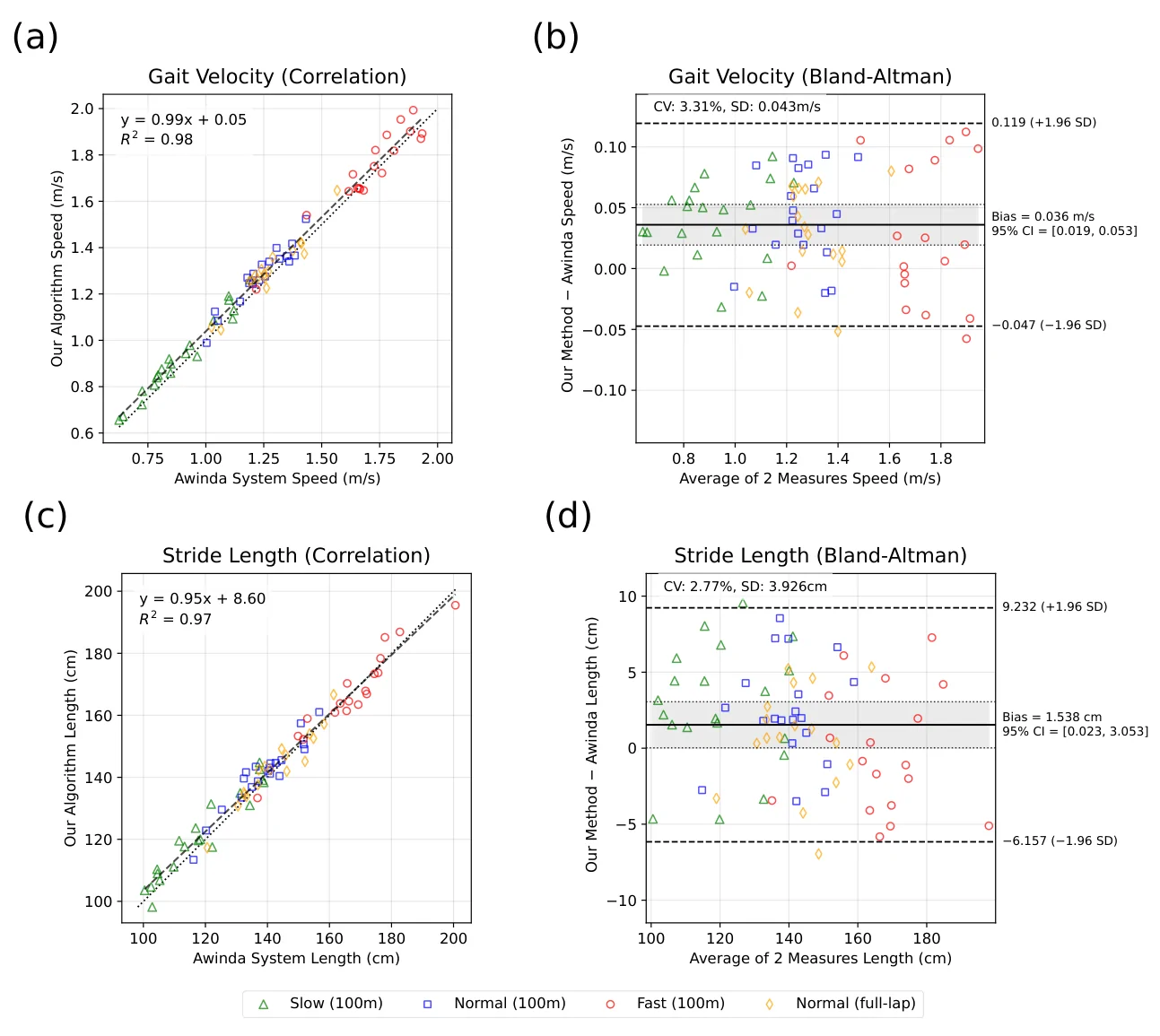
Agreement between our pipeline and the MVN Awinda reference for spatial gait parameters across all trials. Correlation plots are shown in the left column and Bland–Altman plots in the right column. In the correlation plots, the dotted line represents the identity line, and the dashed line represents the fitted regression line. Gait velocity is shown in (**a**,**b**) and stride length in (**c**,**d**).

Across all walking conditions (n=75 bilateral observations), our gait analysis pipeline showed good agreement with the MVN Awinda reference for both temporal (Figure 6) and spatial (Figure 7) parameters. Corresponding descriptive statistics (mean ± SD), task-specific ICCs, and pooled RMSEs are summarized in Table 3. Task-specific ICCs indicated excellent agreement for stride length (0.928–0.961) and stride time (0.989–0.997), good to excellent agreement for gait velocity (0.894–0.957), moderate to excellent agreement for stance time (0.679–0.942), and moderate to good agreement for swing time (0.544–0.790).

The normality assumptions of the linear mixed-effects models were assessed using Q–Q plots of the standardized conditional residuals and estimated participant-level random intercepts (Supplementary Figures S1–S5). The central portions of the conditional-residual distributions broadly followed the reference line for gait velocity, stride length, swing time, and stance time, although some deviations were present in the tails. Stride time exhibited the most pronounced deviations, with a right skew and a heavy upper tail. The estimated participant random intercepts broadly followed the reference line for gait velocity, stride length, stance time, and swing time, whereas stride time showed a marked upper-tail departure. These plots were interpreted as qualitative diagnostics rather than confirmation of normality.

Stride time showed close agreement with the reference system, with an almost unity regression line (slope ≈1.00, $R^{2}\approx 1.00$) and negligible mean bias (0.001 s, 95% CI: [−0.002,0.003] s). In contrast, swing time showed a weaker linear association with MVN ($R^{2}=0.72$, slope =0.73) and wider limits of agreement (LOA: [−0.061,0.051] s; CV =6.22%). The mean bias in swing time was slightly negative (−0.005 s, 95% CI: [−0.015,0.006] s), with the confidence interval including zero.

Gait velocity showed a strong linear association with the reference system ($R^{2}=0.98$, slope =0.99), with a small positive mean bias of 0.036 m/s (95% CI: [0.019,0.053] m/s) and LOA of [−0.047,0.119] m/s (CV =3.31%). Stance time and stride length also showed strong linear associations with MVN Awinda ($R^{2}=0.94$ and 0.97, respectively), with an LOA of [−0.052,0.062] s for stance time (CV =4.34%) and [−6.16,9.23] cm for stride length (CV =2.77%). The mean bias in stance time was small (0.005 s, 95% CI: [−0.005,0.016] s), with the confidence interval including zero, whereas stride length exhibited a positive mean bias of 1.54 cm (95% CI: [0.023,3.053] cm), with the confidence interval excluding zero.

## 4. Discussion

This study evaluated an insole-embedded, foot-mounted nine-axis IMU pipeline for estimating spatiotemporal gait parameters while walking on a 400 m track. A central motivation was to assess whether combining VQF-based orientation estimation with ZUPT-aided error-state Kalman filtering and RTS smoothing can mitigate drift sufficiently to yield reliable gait parameters outside controlled laboratory environments. The system was able to maintain low errors and high agreement with the reference across different walking speeds and path geometries. Across 75 valid trials, our pipeline produced small mean biases and narrow LOAs relative to the MVN Awinda reference system. The ICC point estimates were in the good-to-excellent range for gait velocity, stride length, and stride time; moderate-to-excellent for stance time; and moderate-to-good for swing time. These findings support our hypothesis that our proposed insole-embedded approach is capable of recovering key gait parameters in agreement with an established IMU-based motion capture system in outdoor conditions.

Among the measurements, only the spatial gait parameters exhibited small positive mean biases of 0.036 m/s for gait velocity (95% CI: [0.019, 0.053] m/s) and 1.54 cm for stride length (95% CI: [0.023, 3.053] cm). The confidence intervals for both parameters excluded zero, suggesting systematic positive biases between the two measurement systems. These biases likely reflect small differences in displacement estimation and/or sensor placement between the two systems. However, the velocity bias falls below commonly cited thresholds (0.05 m/s in gait velocity) for meaningful change [56], and the stride length bias corresponds to a small percentage of the mean stride length in our cohort. Although stride time showed negligible mean bias and narrow limits of agreement, the Q–Q plots revealed some deviations from normality in both the conditional residuals and participant random effects. These deviations may affect the reliability of the computed limits of agreement.

The gait velocity ICC was lower during normal 100 m walking than during slow or fast walking, whereas the full-lap ICC was comparable to that of fast walking. The stance time ICCs were lower for both normal walking tasks. These patterns may partly reflect narrower between-participant variability, but the full-lap stance time also showed the largest average between-system difference, so its lower ICC should not be attributed solely to the narrower range. Overall, our error magnitudes were broadly comparable to those reported for foot-worn IMUs and smart insole systems in healthy adults [57,58,59].

Swing time showed lower task-specific ICCs with MVN Awinda (0.544–0.790) than the other temporal parameters across all tasks. However, stance and swing time had the same RMSE (approximately 0.029 s) and showed approximately mirrored biases and limits of agreement, while their sum, stride time, showed minimal errors. This pattern suggests that the disagreement in stance and swing time predominantly arises from differences in TO timing between the two methods. A shift in the detected TO increases the duration of one phase while correspondingly decreasing the other, with little effect on the overall stride time. Consistent with this interpretation, previous work has reported that IMU-based algorithms showed larger errors [26,60] and lower repeatability [26] for temporal parameters that depend on accurate TO detection, such as swing time, than for step or stride time. Many IMU approaches identify gait events using axis-specific peaks or zero crossings in acceleration and/or angular velocity signals and are often developed for specific sensor locations. Therefore, small differences in attachment or orientation can alter these features and degrade event timing, especially for TO [26,61]. This highlights the sensitivity of TO-dependent temporal parameters to the gait event detection accuracy and implementation choices. Additionally, lower between-participant variability relative to the measurement error within individual walking tasks may have contributed to the lower swing time ICCs. Despite the small pooled mean bias of −0.005 s, the regression slope of 0.73 suggests that there might have been proportional bias, with the fitted relationship indicating higher values than MVN Awinda at shorter swing times and lower values at longer swing times. Positive and negative differences may cancel out, producing a small mean bias despite disagreement across the measurement range.

The low mean absolute percentage errors (MAPEs) in the estimated walking distances provide additional support for the spatial estimates of the proposed pipeline, as they demonstrate agreement with independently measured course distances rather than relying solely on comparisons with the MVN Awinda reference system. However, the estimated distance was calculated by integrating the horizontal trajectory and may therefore include additional distance arising from foot motion during swing and deviations from a perfectly straight walking path, potentially leading to slight overestimation. Some variability relative to the measured course distance is also expected because participants did not necessarily stop precisely at the marked finish line, with the final step occurring before, on, or beyond the marker.

Several strengths of this study should be noted. Data were collected on an outdoor 400 m running track environment that approximated real-world predefined trajectory walking, rather than a short laboratory walkway or treadmill. We evaluated multiple walking speeds (slow, normal, and fast) and both straight and curved trajectories. The MVN Awinda system provided a practical reference for this setting. The spatiotemporal gait parameters generated by the MVN Gait Report have previously been validated against optical motion capture [41]. In addition, MVN Awinda has been adopted as a reference motion capture system in previous validation studies of wearable gait and movement analysis methods [62,63]. Together, this prior validation and established use in the literature support its suitability as a practical reference system for the outdoor validation performed in this study.

However, there are also important limitations. Firstly, MVN Awinda is itself an IMU-based motion capture system and does not constitute a fully independent laboratory gold standard. Furthermore, the MVN Gait Report relies on proprietary processing and biomechanical modeling, and some sources of measurement error may be shared with the insole-embedded IMUs evaluated in this study. Agreement should therefore be interpreted as agreement with the selected reference method rather than as a direct measure of absolute accuracy. The ICC estimates should also be interpreted considering the modest task-specific sample sizes (17–20 participants), which limited the precision of some estimates and highlight the need for larger samples in future validation studies. Secondly, the study sample consisted of healthy, mostly younger adults only, and the findings may not directly generalize to older adults, individuals with neurological or musculoskeletal conditions, or those with markedly altered gait patterns. Thirdly, trials were performed on level ground without obstacles or sharp turns; performance in complex indoor environments, on uneven terrain, or during activities such as stair climbing remains to be established. Finally, we performed magnetometer calibration using a standard, basic ellipsoid-fit approach with manual hand calibration. While this method provides a reasonable baseline, it may not be optimally tuned to the specific magnetic characteristics of long-distance experiments, which likely contributed to location-specific trajectory distortions. Therefore, more robust and environment-adaptive magnetometer calibration strategies will be required to achieve lower-error trajectory estimation and to improve consistency across days, locations, and varied real-world environments.

Future work will aim to extend this pipeline to populations with pathological gaits, such as people with Parkinson’s disease or post-stroke hemiparesis, as well as further validation for continuous gait monitoring. Additional validation against an independent laboratory reference, such as optical motion capture, would complement the present outdoor validation and enable a more rigorous assessment of the absolute measurement accuracy. As each participant performed each walking task only once, future studies should also include repeated trials to assess within-participant repeatability. Further studies could also investigate long-term monitoring in free-living environments, integration with additional sensing modalities (e.g., pressure insoles or ultra-wideband-based position references), and further refinement of swing time estimation through enhanced event detection strategies. In parallel, we are actively developing improved magnetometer calibration methods beyond basic ellipsoid fitting to better capture location-specific magnetic distortions and to enable more reliable performance across environments.

Overall, our results indicate that an insole-embedded, foot-mounted nine-axis IMU pipeline with drift mitigation strategies can deliver spatiotemporal gait parameters that closely match those from a reference IMU motion capture system during predefined trajectory walking. These findings support the feasibility of using such systems for gait assessment in outdoor settings in healthy adults and provide a foundation for future work in clinical and home-based monitoring.

## Figures and Tables

**Table 1:** Summary of trial protocols conducted on the 400 m running track at the National University of Singapore. All trials were performed at participant-selected speeds, with no prescribed pacing.

Task ID	Walking Speed	Distance	Trajectory
T1	Normal	100.0 m	Straight
T2	Slow	100.0 m	Straight
T3	Fast	100.0 m	Straight
T4	Normal	437.7 m	Full track lap

**Table 2:** Stride detection summary and estimated walking distance across walking conditions for the 75 retained trials.

Walking Task	*n*	Left Strides (Mean ± SD)	Right Strides (Mean ± SD)	Total Strides	Estimated Distance (m) (Mean ± SD)	Distance MAPE (%)
Slow (100 m)	20	80.45 ± 9.32	78.70 ± 11.32	3183	99.47 ± 2.03	1.08
Normal (100 m)	20	69.15 ± 5.58	68.85 ± 6.26	2760	99.89 ± 0.64	0.55
Fast (100 m)	18	58.11 ± 5.26	57.39 ± 6.03	2079	100.58 ± 2.30	1.20
Normal (full-lap)	17	302.41 ± 25.49	302.24 ± 25.21	10279	437.85 ± 5.34	0.92

*Notes:* Normal (full-lap) denotes the nominal condition name; the measured distance of one complete lap was 437.7 m. Estimated distance is the arithmetic mean of the left- and right-foot trajectory lengths within each trial. MAPE is the mean absolute percentage error of these bilateral trial distance estimates relative to 100 m or 437.7 m, as appropriate.

**Table 3:** Comparison of spatiotemporal gait parameters from our pipeline and MVN Awinda for the 75 retained trials.

Parameter (Units)	Walking Task	Mean Outcome ± SD		ICC	RMSE (Pooled)
		Our Pipeline	MVN Awinda		
Gait velocity (m/s)	Slow (100 m)	0.93±0.17	0.89±0.17	0.957	0.055
	Normal (100 m)	1.28±0.12	1.24±0.12	0.894	
	Fast (100 m)	1.74±0.18	1.71±0.18	0.941	
	Normal (full-lap)	1.30±0.14	1.27±0.13	0.940	
Stride length (cm)	Slow (100 m)	121.20±14.04	118.46±13.61	0.942	4.103
	Normal (100 m)	140.96±10.73	138.59±10.46	0.928	
	Fast (100 m)	167.23±14.27	167.47±14.25	0.961	
	Normal (full-lap)	143.02±11.44	142.37±11.14	0.955	
Stride time (s)	Slow (100 m)	1.32±0.13	1.32±0.13	0.997	0.008
	Normal (100 m)	1.10±0.05	1.11±0.05	0.994	
	Fast (100 m)	0.97±0.06	0.96±0.06	0.989	
	Normal (full-lap)	1.10±0.06	1.10±0.06	0.996	
Stance time (s)	Slow (100 m)	0.82±0.10	0.81±0.09	0.942	0.029
	Normal (100 m)	0.66±0.04	0.66±0.04	0.799	
	Fast (100 m)	0.55±0.05	0.55±0.05	0.850	
	Normal (full-lap)	0.65±0.04	0.64±0.04	0.679	
Swing time (s)	Slow (100 m)	0.51±0.05	0.52±0.05	0.790	0.029
	Normal (100 m)	0.45±0.03	0.45±0.03	0.622	
	Fast (100 m)	0.42±0.02	0.41±0.03	0.655	
	Normal (full-lap)	0.45±0.03	0.46±0.04	0.544	

*Notes:* Task sample sizes are 20, 20, 18, and 17 for slow (100 m), normal (100 m), fast (100 m), and normal full-lap walking, respectively. RMSE = root mean square error, calculated from the 75 paired between-system differences and expressed in the units of the corresponding parameter. ICC denotes the task-specific absolute-agreement, single-measure ICC(A,1).

## Data Availability

The data presented in this study are available on request from the corresponding author. The data are not publicly available because data sharing was not included in the original ethical review (IRB) application or participant consent forms.

## Supplementary Materials

The following supporting information can be downloaded at https://www.mdpi.com/article/10.3390/s26217029/s1. Figure S1: Mixed-effects model diagnostics for gait velocity. Top left: normal Q–Q plot of conditional residuals. Top right: distribution of standardized residuals. Bottom left: standardized residuals versus conditional fitted values. Bottom right: normal Q–Q plot of participant random effects; Figure S2: Mixed-effects model diagnostics for stance time. Top left: normal Q–Q plot of conditional residuals. Top right: distribution of standardized residuals. Bottom left: standardized residuals versus conditional fitted values. Bottom right: normal Q–Q plot of participant random effects; Figure S3: Mixed-effects model diagnostics for stride length. Top left: normal Q–Q plot of conditional residuals. Top right: distribution of standardized residuals. Bottom left: standardized residuals versus conditional fitted values. Bottom right: normal Q–Q plot of participant random effects; Figure S4: Mixed-effects model diagnostics for stride time. Top left: normal Q–Q plot of conditional residuals. Top right: distribution of standardized residuals. Bottom left: standardized residuals versus conditional fitted values. Bottom right: normal Q–Q plot of participant random effects; Figure S5: Mixed-effects model diagnostics for swing time. Top left: normal Q–Q plot of conditional residuals. Top right: distribution of standardized residuals. Bottom left: standardized residuals versus conditional fitted values. Bottom right: normal Q–Q plot of participant random effects.

## Abbreviations

The following abbreviations are used in this manuscript:

IMU	Inertial measurement unit
VQF	Versatile Quaternion Filter
ZUPT	Zero-velocity update
ESKF	Error-state Kalman filter
GPS	Global positioning system
RTS	Rauch–Tung–Striebel
SD	Standard deviation
MCU	Microcontroller unit
TO	Toe-off
HS	Heel strike
CV	Coefficient of variation
LOA	Limits of agreement
ICC	Intraclass correlation coefficient
OLS	Ordinary least squares
$R^{2}$	Coefficient of determination
RMSE	Root mean square error
CI	Confidence interval
REML	Restricted maximum likelihood
MAPE	Mean absolute percentage error
