# Supplementary material for: Sensor Fusion Pipeline for Gait Analysis Using Insole-Embedded Inertial Measurement Units (IMUs)
Source: Sensors (Basel). 2026 Nov 6;26(21):7029. doi: 10.3390/s26217029 (PMC13766370; doi:10.3390/s26217029)
Supplement: Supplementary file 1 [file sensors-26-07029-s001.zip › sensors-4589981-supplementary.pdf]

Supplementary Materials

Sensor Fusion Pipeline for Gait Analysis Using Insole-Embedded Inertial Measurement Units (IMUs)

Supplementary figures

Figures S1–S5 present mixed-effects model diagnostic plots for five gait outcomes. In each figure, red solid lines are Q–Q reference lines, and the red dashed line marks zero standardized residuals.

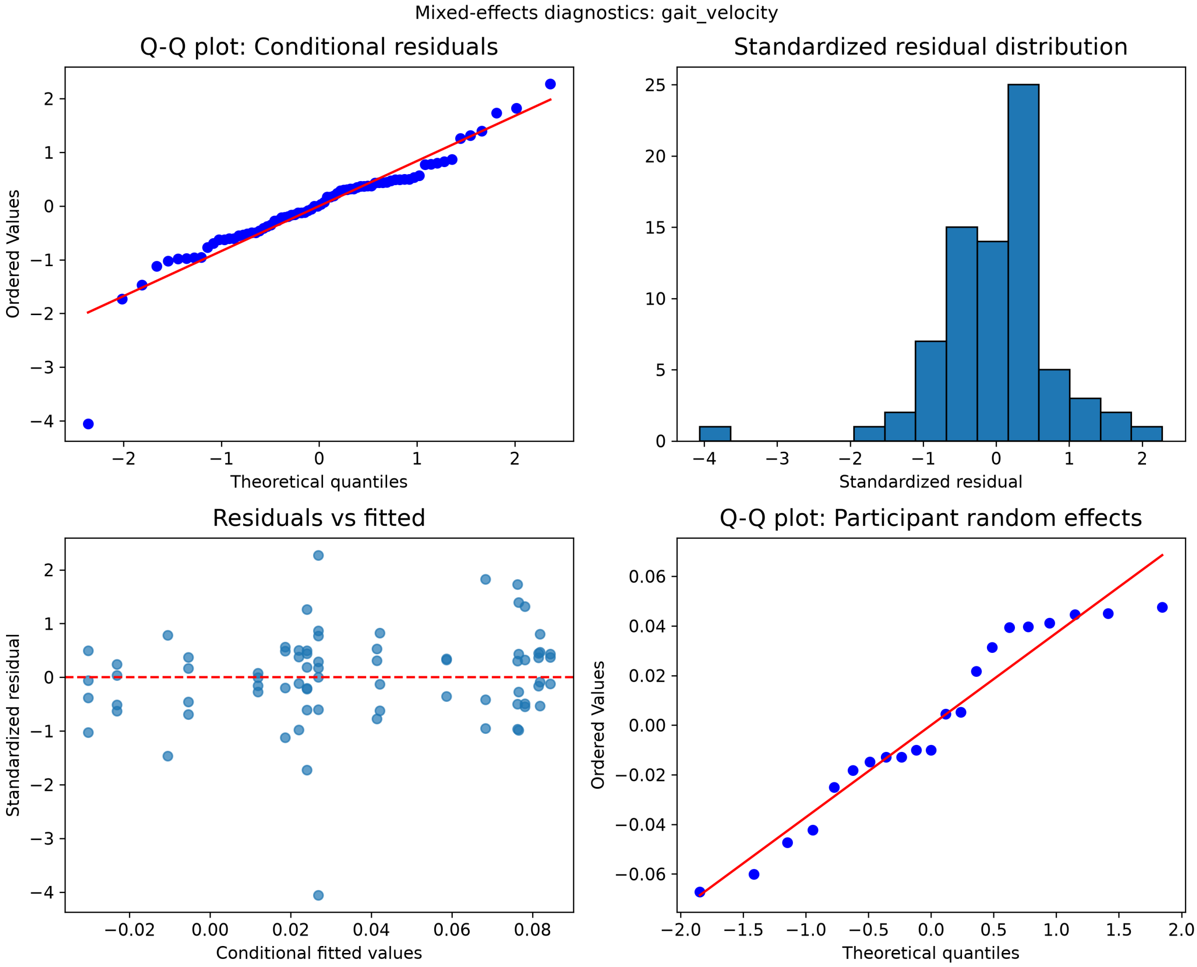

Figure S1: Mixed-effects model diagnostics for gait velocity. Top left: normal Q–Q plot of conditional residuals. Top right: distribution of standardized residuals. Bottom left: standardized residuals versus conditional fitted values. Bottom right: normal Q–Q plot of participant random effects.

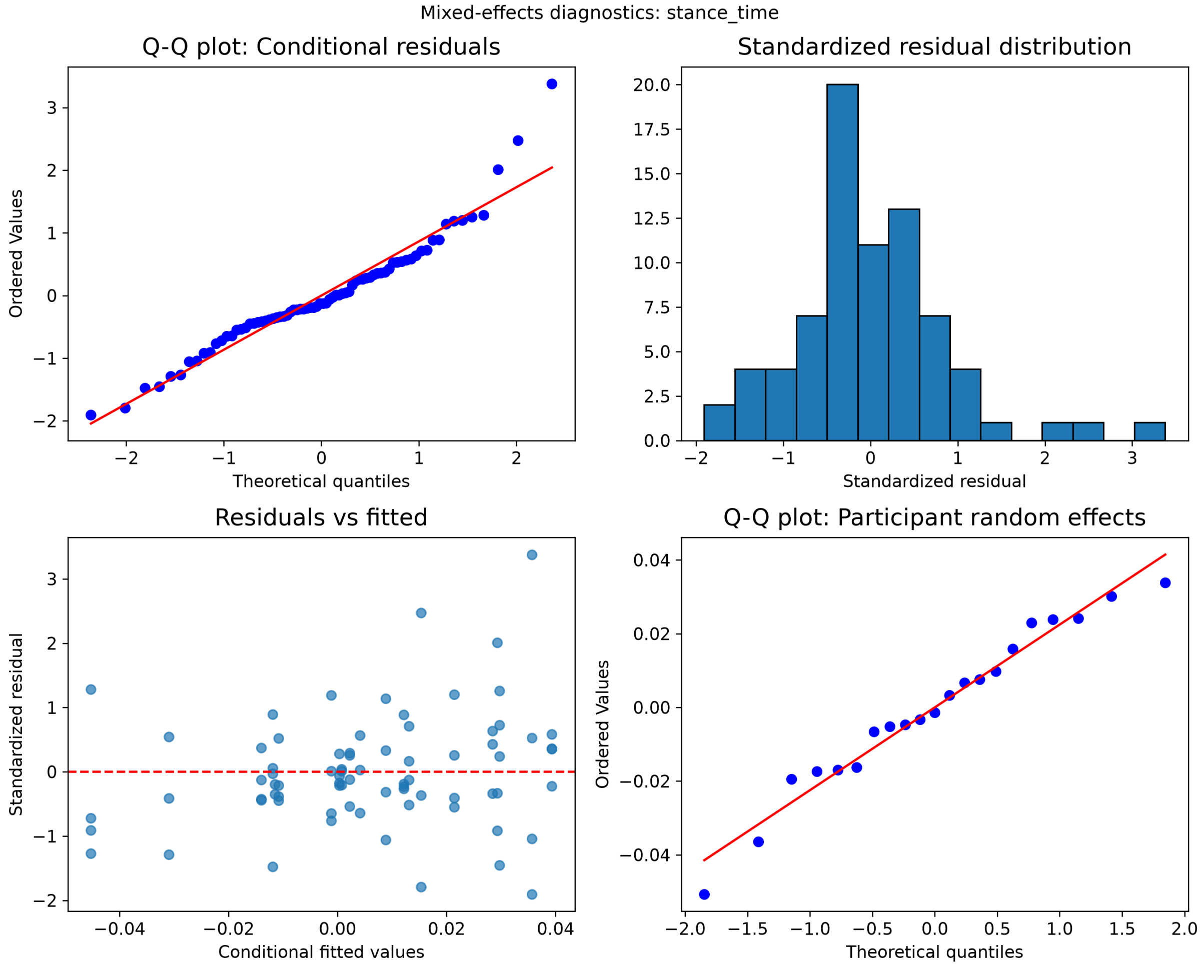

Figure S2: Mixed-effects model diagnostics for stance time. Top left: normal Q–Q plot of conditional residuals. Top right: distribution of standardized residuals. Bottom left: standardized residuals versus conditional fitted values. Bottom right: normal Q–Q plot of participant random effects.

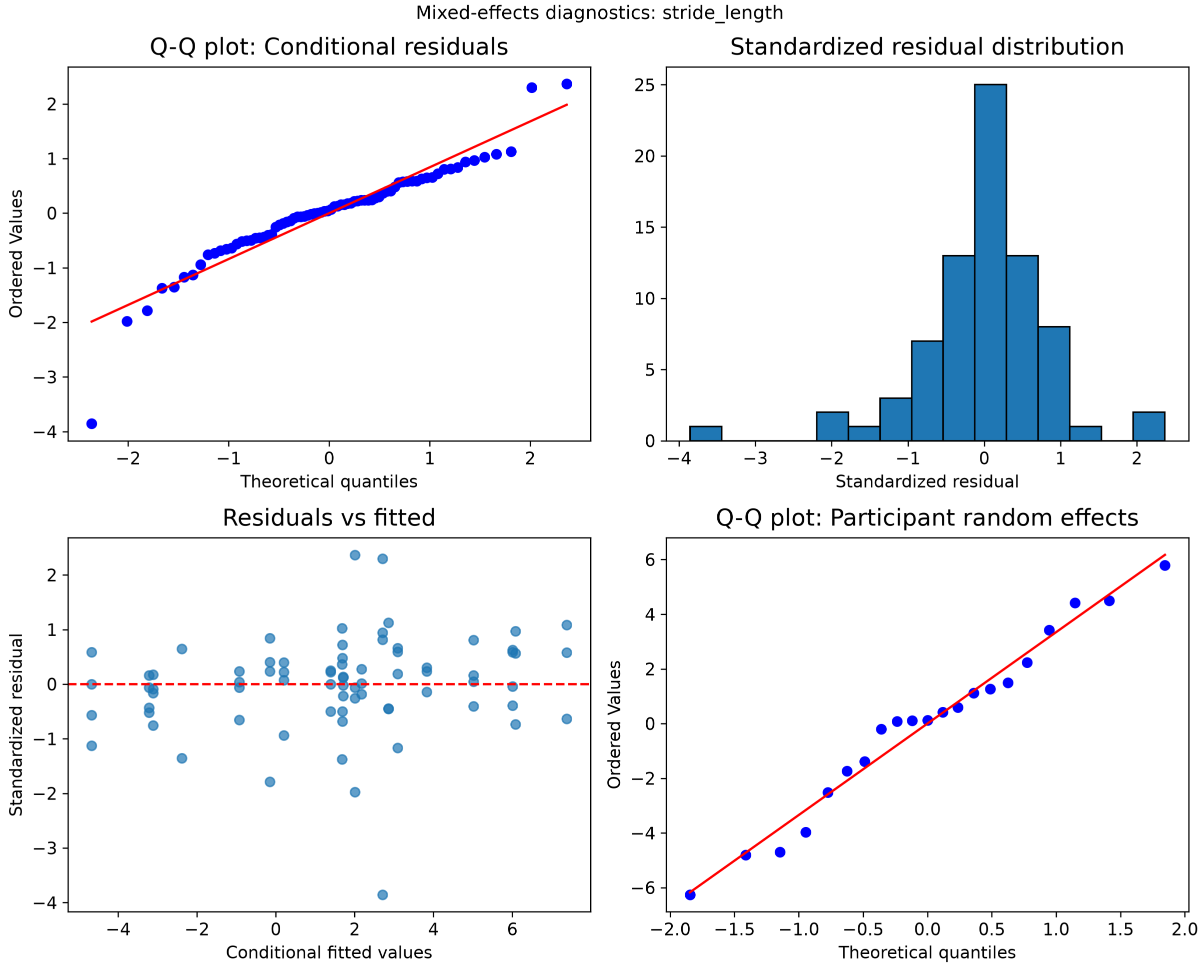

Figure S3: Mixed-effects model diagnostics for stride length. Top left: normal Q–Q plot of conditional residuals. Top right: distribution of standardized residuals. Bottom left: standardized residuals versus conditional fitted values. Bottom right: normal Q–Q plot of participant random effects.

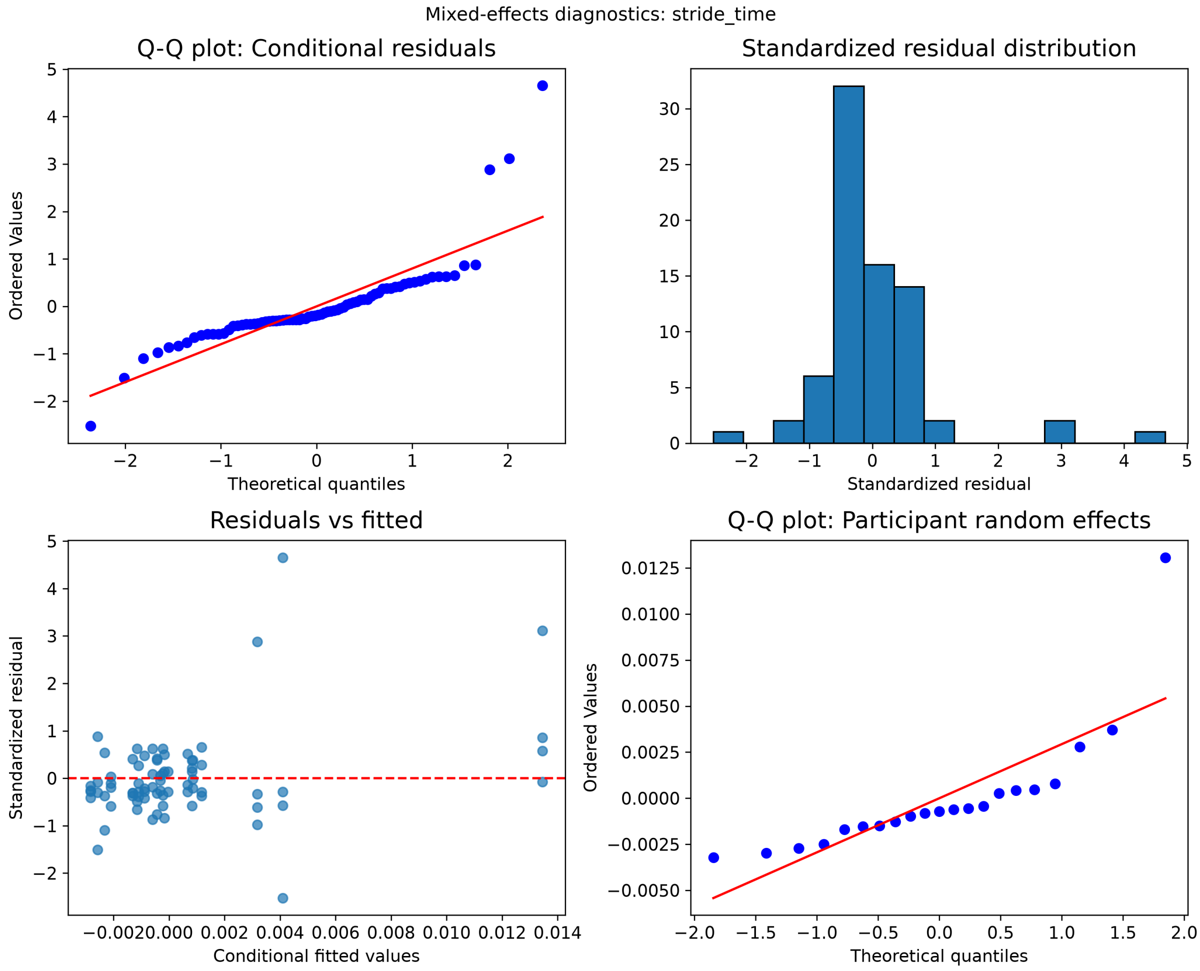

Figure S4: Mixed-effects model diagnostics for stride time. Top left: normal Q–Q plot of conditional residuals. Top right: distribution of standardized residuals. Bottom left: standardized residuals versus conditional fitted values. Bottom right: normal Q–Q plot of participant random effects.

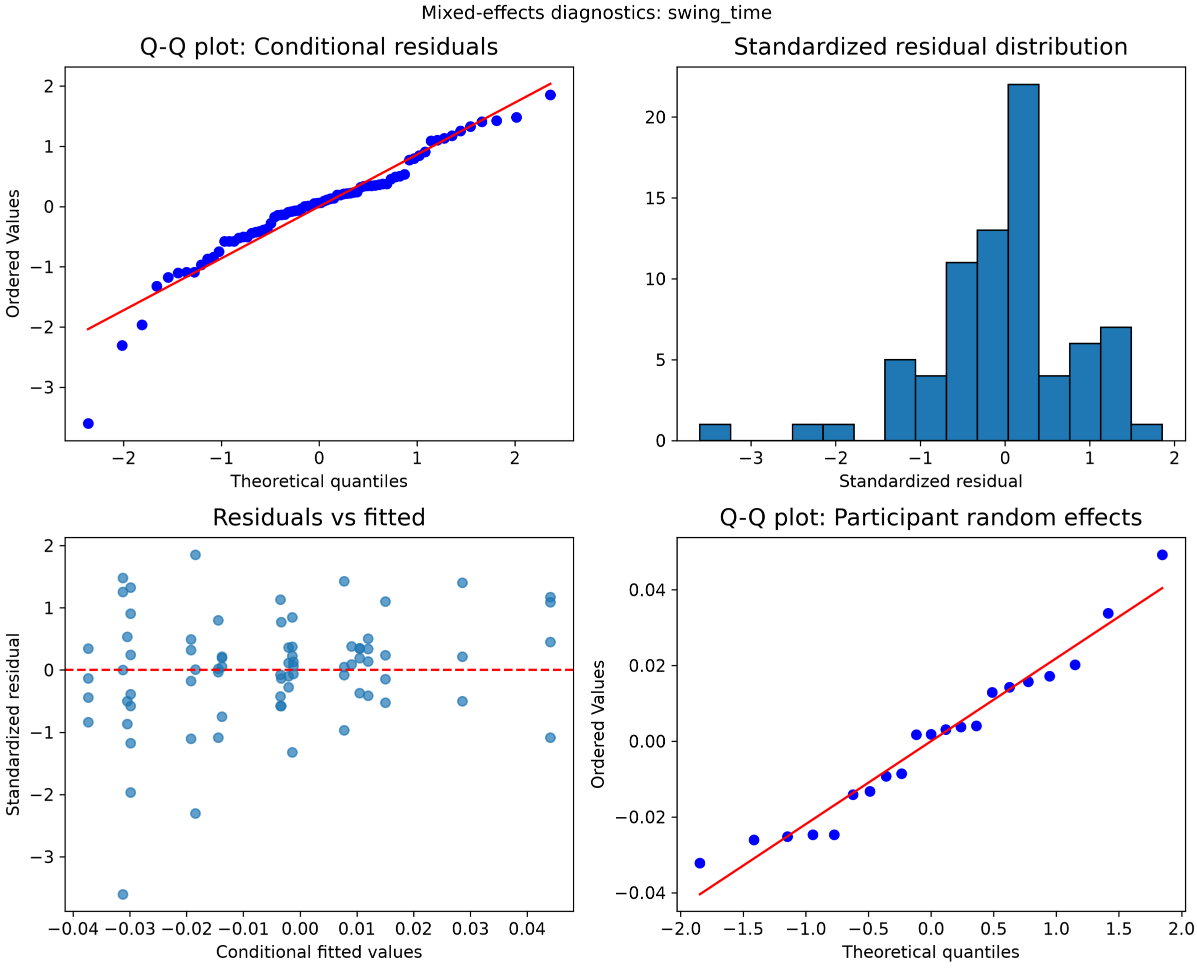

Figure S5: Mixed-effects model diagnostics for swing time. Top left: normal Q–Q plot of conditional residuals. Top right: distribution of standardized residuals. Bottom left: standardized residuals versus conditional fitted values. Bottom right: normal Q–Q plot of participant random effects.
